# Semisynthesis of Selenoauraptene

**DOI:** 10.3390/molecules26092798

**Published:** 2021-05-10

**Authors:** Serena Fiorito, Francesco Epifano, Lorenzo Marchetti, Salvatore Genovese

**Affiliations:** Department of Pharmacy, University “G. d’Annunzio” of Chieti-Pescara, Via dei Vestini 31, 66100 Chieti Scalo, CH, Italy; serena.fiorito@unich.it (S.F.); marchetti_lorenzo@libero.it (L.M.); s.genovese@unich.it (S.G.)

**Keywords:** auraptene, 7-geranylselenocoumarin, Newman-Kwart rearrangement, selenium compounds, selenoauraptene, selenoprenylcoumarins

## Abstract

Selenium-containing compounds are gaining more and more interest due to their valuable and promising pharmacological properties, mainly as anticancer and antioxidant agents. Ebselen, the up to now only approved drugs, is well known to possess very good glutathione peroxidase mimicking effects. To date, the most of efforts have been directed to build pure synthetic Se containing molecules, while less attention have been devoted to Se-based semisynthetic products resembling natural compounds like terpenes, polyphenols, and alkaloids. The aim of this short communication is to report the synthesis of the first example of a Se-phenylpropanoids, namely selenoauraptene, containing a selenogeranyl side chain in position 7 of the umbelliferone core. The key step was the Newman-Kwart rearrangement to obtain a selenocarbamate in which the Se atom was directly attached to umbelliferone (replacing its 7-OH function) followed by hydrolysis to get diumbelliferyl diselenide, which was finally easily converted to the desired Se-geranyl derivative in quite a good overall yield (28.5%). The synthesized adduct displayed a greater antioxidant and a radical scavenger in vitro activity than parent auraptene. The procedure we describe herein, to the best of our knowledge for the first time in the literature, represents an easy-to-handle method for the synthesis of a wide array of seleno analogues of naturally occurring biologically active oxyprenylated secondary metabolites.

## 1. Introduction

**Figure 1:**
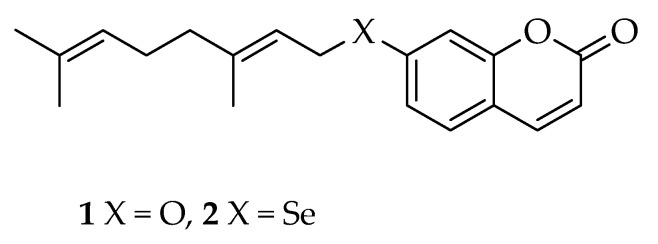
Structure of auraptene (**1**) and selenoauraptene (**2**).

During the last decade, rare natural products have been the subject of intensive research, with the objective of better depicting their pharmacological profile [1]. Among the best investigated classes in this regard over the last two decades are oxyprenylated secondary metabolites of plant, fungal, and microbial origin. These are phytochemicals of mixed biosynthetic origin and include compounds possessing different skeletons such as phenylpropanoids, polyketides, and alkaloids with attached hemiterpenyl, monoterpenyl, and, less frequently, sesquiterpenyl side chains. The first group have been the subject of more attention concerning their chemical and pharmacological properties. What is noteworthy about oxyprenylated phenylpropanoids is their capacity to act as dietary feeding chemopreventive agents of acute and chronic syndromes affecting humans, such as cancer, inflammation, stroke, neurological disorders, and several others [2]. The majority of review and research articles that have appeared in the literature in recent years have focused on selected oxyprenylated secondary metabolites, like auraptene **1** (7-geranyloxycoumarin, Figure 1) [3].

Auraptene is the most abundant geranyloxycoumarin found in nature, and its plant sources and pharmacological properties were reviewed for the first time in 2011, and subsequently in 2020 [4,5]. Its presence has been documented in several edible fruits and vegetables, like *Citrus* spp. fruits and pomegranate. The reported pharmacological properties for auraptene include growth inhibitory activity and tumor suppression activities in vitro and, more notably, in vivo against a wide panel of cancers (e.g., skin, tongue, stomach, colon, lung, prostate, and breast), immunomodulatory effects, anti-inflammatory capacities, modulation of lipid and sugar metabolism, and finally neuroprotective properties. Auraptene has a pleiotropic mode of action, being able to trigger several putative biomolecular targets [2,3,4,5]. From a medicinal chemistry point of view, several structural analogues have been extracted from natural sources and/or synthesized. The modifications of the basic skeleton of auraptene to yield similarly biologically active compounds include substitution of the α,β-unsaturated lactone ring with α,β-unsaturated carboxylic acid [6] and α,β-unsaturated aldehyde moieties [7], introduction of methoxy groups, halogens, and additional terpenyl chains as substituents of the aromatic ring [8,9,10], functionalization of the geranyloxy side chain [11,12], and finally introduction of different substituents in the lactone portion [13,14]. The plethora of literature data related to this suggests that the synthesis and investigation of the pharmacological properties of isosters and structural analogues of auraptene is a field of current and active research with many potential outcomes and practical uses in the near future. None of the natural and semisynthetic compounds investigated up until now have been derived from a replacement of the ether oxygen atom in position 7 of the umbelliferone ring. Thus, we decided to conceive and set up the chemical semisynthesis of the first semisynthetic derivative of auraptene, namely selenoauraptene **2** (7-geranylselenoumbelliferone, Figure 1), containing a chalcogen atom belonging to group 16 of the periodic table of the elements, like oxygen. Such an approach is unprecedented in the literature, to the best of our knowledge. The topic of this short communication is the description of the chemical semisynthesis of selenoauraptene **2**.

## 2. Results and Discussion

**Figure 2:**
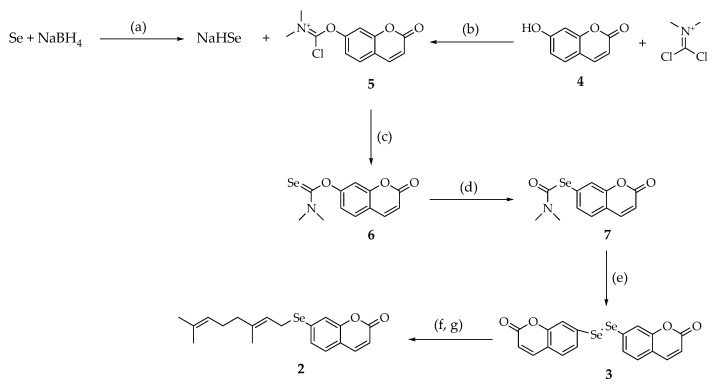
Semisynthesis of selenoauraptene **2.** Reagents and conditions: (a) 2:1 molar ratio of NaBH_4_ to powdered gray Se, iPrOH, 1 h, 5 °C; (b) CH_2_Cl_2_, 45 °C, 24 h; (c) iPrOH, 1.5 h, r.t., column chromatography; (d) neat, 200 °C; (e) KOH, MeOH, H_2_O, 24 h, r.t. (f) NaBH_4_ (5 equiv.), iPrOH, geranyl bromide (1.1 equiv.), 1 h, r.t.; (g) crystallization (n-hexane).

Up until now, methods for the preparation of selenophenols have commonlybeen based on harsh chemical processes such as Grignard chemistry [15], nucleophilic-aromatic-type substitutions [16], or Sandmeyer-type chemistry [17]. The Newman-Kwart rearrangement is an effective and valid reaction in which the aryl group of an *O*-aryl thiocarbamate, ArOC(=S)NMe_2_, migrates from the oxygen atom to the sulfur atom, forming an *S*-aryl thiocarbamate, ArSC(=O)NMe_2_ [18]. The recent discovery that such a rearrangement can also be applied without significant differences to *O-*aryl selenocarbamate ArOC(=Se)NMe_2_ [19] prompted us to explore the possibility of introducing a selenium atom replacing oxygen in a phenylpropanoid core, finally providing the hitherto undescribed diumbelliferyl diselenide **3**. To this aim, based on our previous experience in handling this natural compound, we selected umbelliferone **4** as the substrate. The experimental protocol we set up enabled the preparation of the desired diselenide from the mentioned coumarin in three easy-to-handle steps (Figure 2).

**Figure 3:**
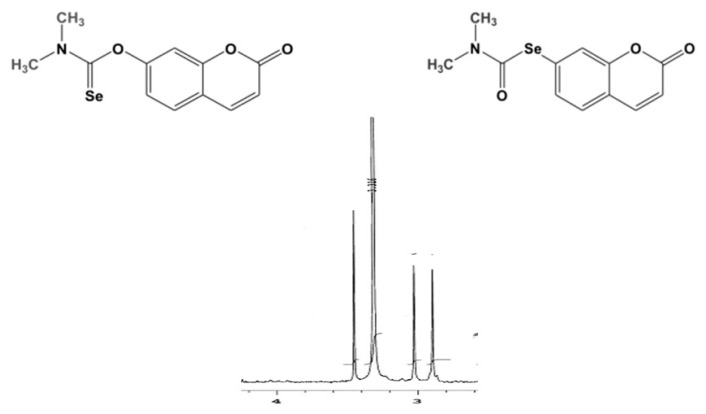
Monitoring the conversion of O-aryl selenocarbamate **6** into the Se-aryl carbamate **7** by ^1^H NMR.

Adopting a synthetic route similar to that reported in the literature by Sørensen and coworkers in 2013 [19], the first reaction we performed consisted of the activation of the phenol moiety of umbelliferone by its reaction with commercially available *N*-(dichloromethylene)-*N*-methylmethanaminium chloride in a 1.1/1 equiv. ratio in refluxing CH_2_Cl_2_ for 24 h. This step allowed the in situ formation of the intermediate **5**. The resulting solution was added dropwise over a period of 30 min to a mixture containing NaHSe, in turn generated from the reduction of elemental Se with NaBH_4_ in iPrOH as the solvent for 1 h at 5 °C. The progress of this last process was monitored by the complete decoloration of the suspension containing elemental Se. After mixing, the overall solution was further kept under vigorous magnetic stirring at r.t. under N_2_ for 90 min. The reaction was monitored by TLC. After filtration on celite to remove the unreacted elemental Se, the filtrate was evaporated under vacuum to dryness. The crude product obtained was then purified by SiO_2_ gel column chromatography, using a 99.5:0.5 CH_2_Cl_2_/MeOH as the eluent, and the desired adduct **6** was isolated as a yellow solid with a yield of 42%. The following step, the Se-Newman-Kwart rearrangement of the *O*-aryl selenocarbamate **6**, was first performed by applying microwave (MW) irradiation in solvent-free conditions. Under these experimental conditions and MW heating using different power values in the range 200 W–400 W and different reaction times, from 10 min up to 60 min, no conversion was observed, and the formation of numerous byproducts, which were difficult to separate by column chromatography and which did not include the desired one, was detected by TLC. While it has been reported that the “classic” Newman-Kwart rearrangement can effectively proceed under microwave irradiation [20], the corresponding “seleno” version did not find any application, probably because it is very hard to tune the potency of the microwave apparatus with the risk to expose selenoderivatives to excessive heating, and consequently to oxidation and chemical degradation in general, as we observed in our experiments. To this end, very recently, Kundu reported that the scope of transition metal catalyst-free processes for the synthesis of organoselenides under microwave irradiation is still very poor [21]. Thus, we decided to carry out the same process again with no solvents, but under conventional heating (200 °C). Thus, after 1 h and monitoring the progress of the reaction by thin layer chromatography (TLC), a complete conversion of adduct **6** into a slightly more polar product was observed. After purification by SiO_2_ gel column chromatography using the same mixture as mentioned above as the mobile phase, the Se-aryl carbamate **7** was obtained in 87% yield as a yellowish solid. The structure of this adduct was fully characterized by mass spectrometry (MS) and NMR spectroscopy. In subsequent experiments leading to the same adduct, we more successfully and effectively monitored the progress of the reaction by ^1^H NMR of samples taken at different times from the reaction vessel. As outlined in Figure 3, the most diagnostic signals are represented by the two methyl groups attached to the nitrogen atom, the chemical shift of which are decreased from 3.45 ppm and 3.28 ppm to 3.03 ppm and 2.90 ppm, respectively.

Such a monitoring technique was seen to be more powerful and more versatile than TLC to effectively follow the progress of the very crucial step of the overall synthetic route to selenoauraptene. 

Se-aryl carbamate **7** was then hydrolized using a 20% KOH H_2_O/MeOH 1:1 solution at r.t. After 24 h, the diumbelliferyl diselenide **3** was obtained as a yellow solid in 99% yield. To accomplish the final step finalizing the semisynthesis of selenoauraptene **2**, compound **3** was dissolved in iPrOH and solid NaBH_4_ (5 equiv.) was added to the solution until a complete decoloration was achieved. After 5 min, geranyl bromide was added in small aliquots and the resulting mixture was allowed to react at r.t. for 1 h. After acid–base work-up, the crude product was purified by crystallization (*n*-hexane) providing selenoauraptene **2** in 78% yield as a pale yellow solid. The structure of the final adduct has been confirmed by GC/MS and NMR spectroscopy. The overall yield of compound **2** *was 28.5*%.

Organoselenium compounds are nowadays well recognized to play important roles in a wide range of chemistry, biochemistry, and materials chemistry applications. Selenium is typically present in biomolecules like selenocysteine and selenomethionine. The first is a key component in the active of several enzymes modulating redox cell cycles in human body like glutathione peroxidase (GPx) [22]. Selenomethionine also exhibits valuable pharmacological properties such as antioxidant catalytic activity and as an anticancer agent [23]. Organoselenium derivatives have frequently been applied as synthetic mimetic of GPx. Ebselen is the main example of a synthetic organoselenium compound recently introduced into common therapeutic practice, having a marked GPx-like activity. It is a multifunctional compound, which catalyzes several essential reactions for the protection of cellular components from oxidative and free radical damage [24]. Thus, the search for novel and alternative organoselenium compound with enhanced and more selective pharmacological activities is a field of current and growing interest [25]. In this context, we described herein the semisynthesis of the first semisynthetic Se-containing derivative of auraptene **1**. Such an approach is unprecedented in the literature to the best of our knowledge. Mere structural considerations make it possible to hypothesize that selenoauraptene may have the same or an enhanced potential from a pharmacological point of view with respect to the parent auraptene. The putative pharmacophore of compound **2** may be seen as split into three regions, all representing an ideal base to confer to the overall molecule a wide array of capacities to interact with and modulate several biological targets. First, the geranyl side chain is nowadays well known to trigger numerous cell structure like enzymes, membranes and nuclear receptors, having a deep modulatory role on their function [26]. Indeed, prenyl chains have been selected from an evolutionary point of view as key determinants in the functions of a plethora of cell substructures, from bacteria to eukaryotes, that comprise mammals [27]. Secondly the coumarin nucleus is suggested by numerous literature data to be a versatile scaffold in drug design and discovery and a structural determinant for pharmacological activities [28]. Finally, the selenium atom, bridging the aforementioned moieties, may contribute to the overall effects thanks to the known activities (e.g., antioxidant) ascribed in the literature to selenoethers [29]. Consequently, it may be hypothesized that selenoauraptene **2** and other semisynthetic selenoprenylated secondary metabolites that could hopefully be obtained in the near future, using naturally occurring compounds as templates, may represent a novel and alternative class of biologically active agents with a great potential. In this regard, a preliminary assay regarding the antioxidant and radical scavenger activities of the obtained semisynthetic product was carried out using the 2,2-diphenyl-1-picrylhydrazyl (DPPH) and 2,2-azino-bis(3-ethylbenzothiazoline-6-sulfonic) acid diammonium salt (ABTS) using auraptene for comparison and ascorbic acid and Trolox^®^ used as the reference [30,31,32,33]. The results of this assay are reported in Table 1.

From data reported in Table 1, it is highly evident that the selenoauraptene we obtained is characterized by appreciable to very good dose-dependent antioxidant and radical scavenger effects, greatly higher than the parent natural product auraptene, and comparable to and slightly higher than the known antioxidants ascorbic acid and Trolox^®^, which were used as the references. These very preliminary data are extremely encouraging for accomplishing further assays to reveal the effective pharmacological properties of selenoaurapteneare and other coumarin selenoethers that will be synthesized in the near future. Such experiments are now being carried out in our laboratory.

## 3. Materials and Methods

General information. Reactions were carried out in round-bottom flasks and were stirred with Teflon-coated magnetic stirring bars. All reagents and solvents were obtained from commercial sources and were used as received unless otherwise stated. Reactions were routinely monitored by TLC using Merck silica gel F_254_ plates and visualized by UV irradiation (254 nm) or by KMnO_4_ staining. Silica gel Kieselgel 60 (70–230 mesh, Merck, Darmastadt, Germany) was used for column chromatography. NMR experiments were carried out at 25 °C with a Varian 300 spectrometer (Bruker, Milan, Italy) operating at 300 MHz for ^1^H, 75.45 MHz for ^13^C. ^1^H and ^13^C chemical shifts (δ) are reported in parts per million (ppm), relative to TMS (δ = 0.0 ppm) and the residual solvent peak of CDCl_3_ (δ = 7.26 and 77.00 ppm in ^1^H and ^13^C-NMR, respectively). DPPH and ABTS assays have been carried out as already described in the literature [25].

*O-(2-Oxo-2H-chromen-7-yl) dimethylcarbamoselenoate***6**. To a solution of umbellic. ferone **4** (0.187 g, 1.1 mmol) in CH_2_Cl_2_ was added *N*-(dichloromethylene)-*N*-methylmethanaminium chloride (0.162 g, 1.0 mmol) and the resulting mixture was refluxed under magnetic stirring for 24 h. In a separate vessel, to a suspension of elemental selenium (0.1 g, 1.2 mmol) and NaBH_4_ (0.09 g, 2.4 mmol) in *i*PrOH (4 mL) under a N_2_ atmosphere and a vigorous magnetic stirring was allowed to react until a colorless solution was obtained. This latter was then added dropwise along a period of 30 min. to the solution containing compound **5** and the resulting mixture was further kept under vigorous magnetic stirring at r.t. under N_2_ for 90 min. After filtration on celite, the filtrate was evaporated to dryness under vacuum. The crude product so obtained was then purified by SiO_2_ gel column chromatography (eluent CH_2_Cl_2_/MeOH). Compound **6** was obtained as a yellow solid. Yield 42%; m. p. 137–139 °C (dec.); IR 1535 (s), 1610 (s) cm^−1^; ^1^H NMR δ 3.28 (s, 3H), 3.45 (s, 3H), 6.22 (d, 1H, *J* = 9.2 Hz), 6.71 (s, 1H), 6.88 (d, 1H, *J* = 4.8 Hz), 7.56 (d, 1H, *J* = 4.8 Hz), 7.87 (d, 1H, *J* = 9.2 Hz); ^13^C NMR δ 18.9, 22.1, 103.2, 112.0, 112.4, 124.3, 128.0, 142.2, 154.1, 156.7, 157.6, 162.1; HRMS (m/z) 296.9902 (100%), 294.9912 (47%), 292.9930 (19%). Anal calcd. for C_12_H_11_NO_3_Se: C, 48.66; H, 3.74; N, 4.73; O, 16.21; Se, 26.66. Found: C, 48.61; H, 3.71; N, 4.68; O, 16.16; Se, 26.62.

*2-Oxo-2H-chromen-7-yl dimethylselenocarbamate***7**. *O*-(2-Oxo-2*H*-chromen-7-yl) dimethylcarbamoselenoate **6** (300 mg, 1.01 mmol) was put in a round-bottom flask and heated in an oil bath at 200 °C for 1 h under a N_2_ atmosphere, monitoring the progress of the reaction by ^1^H NMR. The crude product so obtained was then purified by SiO_2_ gel column chromatography (eluent CH_2_Cl_2_/MeOH) providing compound **7** as a yellowish solid. Yield 87%; m. p. 155–158 °C (dec.); IR 1590 (s), 1610 (s) cm^−1^; ^1^H NMR δ 3.03 (s, 3H), 2.90 (s, 3H), 6.28 (d, 1H, *J* = 9.3 Hz), 6.75 (s, 1H), 6.92 (d, 1H, *J* = 4.6 Hz), 7.48 (d, 1H, *J* = 4.6 Hz), 7.72 (d, 1H, *J* = 9.3 Hz); ^13^C NMR δ 19.2, 21.0, 103.2, 112.0, 112.4, 124.3, 128.0, 142.2, 148.2, 155.2, 159.7, 162.9; HRMS (m/z) 296.9904 (100%), 294.9908 (49%), 292.9926 (22%). Anal calcd. for C_12_H_11_NO_3_Se: C, 48.66; H, 3.74; N, 4.73; O, 16.21; Se, 26.66. Found: C, 48.59; H, 3.79; N, 4.77; O, 16.15; Se, 26.59. 

*Diumbelliferyl diselenide***3**. 2-Oxo-2*H*-chromen-7-yl dimethylselenocarbamate **7** (180 mg, 0.606 mmol) was dissolved in a 20% KOH H_2_O/MeOH 1:1 solution (3 mL) and the resulting mixture was allowed to react at r.t. for 24 h. The solution was diluted with H2O (20 mL), the pH adjusted to 2.0 by addition of HCl 37%, and finally extracted with *n*-hexane (3 × 10 mL). The collected organic phases were dried over Na_2_SO_4_, and the solvent evaporated to dryness under vacuum, providing the desired product **3** as a brilliant yellow solid. Yield 99%; m. p. 86–88 °C (dec.); IR 1615 (s) cm^−1^; ^1^H NMR δ 6.28 (d, 1H, *J* = 9.2 Hz), 6.70 (s, 1H), 6.92 (d, 1H, *J* = 4.6 Hz), 7.44 (d, 1H, *J* = 4.6 Hz), 7.83 (d, 1H, *J* = 9.2 Hz); ^13^C NMR δ 103.2, 112.0, 112.4, 124.3, 128.0, 142.2, 144.9, 152.1, 161.9; HRMS (m/z) 449.8906 (100%), 447.8910 (38%), 445.8930 (19%). Anal calcd. for C_18_H_10_O_4_Se_2_: C, 48.24; H, 2.25; O, 14.28; Se, 35.24 Found: C, 48.18; H, 2.22; O, 14.21; Se, 35.24.

*Selenoauraptene***2**. Diumbelliferyl diselenide **3** (150 mg, 0334 mmol) was dissolved in *i*PrOH and solid NaBH_4_ (63 mg, 1.67 mmol) was added to the solution in small aliquots until a complete decoloration was observed. After 5 min geranyl bromide (76 mg, 0.35 mmol) was added dropwise and the resulting mixture was allowed to react at r.t. for 1 h. After acid–base work-up, the crude product was purified by crystallization (n-hexane) providing the desired compound **2** as a pale-yellow solid. Yield 87%; m. p. 72–74 °C; IR 1610 (s) cm^−1^; ^1^H NMR δ 1.58 (s, 3H), 1.62 (s, 3H), 1.72 (s, 3H), 2.04–2.21 (m, 4H), 3.84 (m, 2H), 4.94 (m, 1H), 5.23 (m, 1H), 6.29 (d, 1H, *J* = 9.3 Hz), 6.83 (s, 1H), 6.88 (d, 1H, *J* = 4.4 Hz), 7.39 (d, 1H, *J* = 4.4 Hz), 7.71 (d, 1H, *J* = 9.3 Hz); ^13^C NMR δ 15.6, 18.3, 21.2, 28.4, 29.1, 55.4, 103.1, 108.9, 110.2, 111.9, 113.0, 125.1, 128.4, 139.8, 140.3, 141.9, 145.1, 151.3, 160.8; HRMS (m/z) 449.8906 (100%), 447.8910 (38%), 445.8930 (19%). Anal calcd. for C_18_H_10_O_4_Se_2_: C, 48.24; H, 2.25; O, 14.28; Se, 35.24 Found: C, 48.18; H, 2.22; O, 14.21; Se, 35.24.

DPPH assay. General procedure. Selenoauraptene was tested at three concentration levels: 10 µM, 50 µM, and 100 µM. Auraptene and ascorbic acid, used as the references, were assayed at the same values. A 150 µL amount of the solution of the compound under investigation was mixed with the same volume of a solution of DPPH free radical 0.04 mg/mL. The reaction was monitored after 30 min. Absorbance at 517 nm was recorded by a Varian Cary^®^ 50 UV-Vis spectrophotometer and used to calculate radical scavenging activity (% of inhibition) with the Equation (1):

Inhibition (%) = 1 − (Abs_sample_ − Abs_blank/_Abs_control_ − Abs_blank_) × 100 (1)

where Abs_sample_ was the absorbance of the reaction in presence of sample (sample dilution + DPPH free radical solution), Abs_blank_ was the absorbance of the blank for each sample dilution (sample dilution + DPPH free radical solution) and Abs_control_ was the absorbance of control reaction (sample solvent + DPPH free radical solution). All determinations were performed in triplicate (*n* = 3).

*ABTS assay*. *General procedure.* The stock solutions included 7 mM ABTS solution and 2.4 mM potassium persulfate solution. The working solution was then prepared by mixing the two stock solutions in equal quantities and allowing them to react for 14 h at room temperature in the dark. The solution was then diluted by mixing 1 mL ABTS solution with 60 mL MeOH to obtain an absorbance of 0.706 ± 0.01 units at 734 nm using a Varian Cary^®^ 50 UV-Vis spectrophotometer. Fresh ABTS solution was prepared for each assay. Synthesized and references compound solutions at the same concentration as above (1 mL) were allowed to react with 1 mL of the ABTS solution and the absorbance was recorded at 734 nm after 7 min using the same apparatus as above. The ABTS scavenging capacity of the extract was compared with that of Trolox^®^ and percentage inhibition calculated as ABTS radical scavenging activity following the Equation (2):

ABTS radical scavenging activity = (Abs_control_ − Abs_sample/_Abs_control_) × 100 (2)

where Abs_control_ is the absorbance of ABTS free radical in MeOH; Abs_sample_ is the absorbance of ABTS free radical solution mixed with sample compounds/reference. All determinations were performed in triplicate (*n* = 3).

## Figures and Tables

**Table 1:** Antioxidant activities of selenoauraptene by the DPPH and ABTS assays.

	% Antioxidant Activity *		
**DPPH**	**10 µM**	**50 µM**	**100 µM**
Selenoauraptene	44.6 ± 0.12	72.5 ± 0.06	93.1 ± 0.12
Auraptene	21.5 ± 0.04	39.6 ± 0.08	61.1 ± 0.04
Ascorbic acid	45.1 ± 0.09	68.1 ± 0.09	85.9 ± 0.07
**ABTS**			
Selenoauraptene	51.3 ± 0.07	71.8 ± 0.04	91.1 ± 0.09
Auraptene	19.7 ± 0.03	29.9 ± 0.05	49.9 ± 0.06
Trolox^®^	44.2 ± 0.09	69.8 ± 0.06	88.2 ± 0.08

* Values are shown as the mean ± S.D. of three independent experiments.

## Data Availability

Data is contained within the article.

## Sample Availability

Samples of the compounds are available from the authors.
